# Frequency Specificity of fMRI in Mesial Temporal Lobe Epilepsy

**DOI:** 10.1371/journal.pone.0157342

**Published:** 2016-06-17

**Authors:** Shuyan Song, Mingyue Ding, Hong Li, Xiaopeng Song, Wenliang Fan, Xuming Zhang, Haibo Xu

**Affiliations:** 1 School of Life Science and Technology, Key Laboratory of Image Processing and Intelligent Control of the Ministry of Education, Key Laboratory of Molecular Biophysics of the Ministry of Education, Huazhong University of Science and Technology, Wuhan, China; 2 Department of Radiology, The Second Affiliated Hospital of Zhejiang University College of Medicine, Hangzhou, China; 3 Department of Biomedical Engineering, College of Engineering, Peking University, Beijing, China; 4 Department of Radiology, Union Hospital, Tongji Medical College, Huazhong University of Science and Technology, Wuhan, China; 5 Department of Radiology, Zhongnan Hospital of Wuhan University, Wuhan, China; University of Modena and Reggio Emilia, ITALY

## Abstract

Medial temporal lobe epilepsy (mTLE) is a system-level disease characterized by aberrant neuronal synchronization and widespread alterations in function. Previous studies have focused on the amplitude analysis of the blood oxygenation level-dependent (BOLD) signals to reveal the aberrant alterations in mTLE. However, these methods did not work well in the cases where the amplitudes of two oscillations are correlated but the underlying oscillations are neither phase coherent nor frequency consistent. To address this problem, we investigated the differences of frequency specificity between patients with mTLE and healthy controls using the extreme-point symmetric mode decomposition (ESMD) method. In this method, the BOLD signals were decomposed into a set of intrinsic mode functions (IMFs) and the instantaneous frequency of each IMF was calculated using the direct interpolation strategy. The intrinsic frequency (denoted as *Freq*) for every voxel was obtained by the weighted sum of the instantaneous frequencies of all the IMFs. The *Freq* was used as an index to evaluate the altered frequency specificity of 41 patients with mTLE (17 right-side, 24 left-side) and 24 healthy control subjects. The results show that the peak of frequency distribution curve for the patients moves towards the higher frequency than that for the healthy controls. Compared with the healthy control group, the patients with left mTLE demonstrate higher *Freq* in the default mode network, middle frontal gyrus, insula, middle temporal gyrus and calcarine gyrus; the patients with right mTLE demonstrate higher *Freq* in the precuneus and occipital lobe. For the three groups, the distinct frequency distribution appears in the left and right hippocampus due to the hippocampal structural and functional asymmetries. The preliminary results imply that the frequency-specific correlated oscillations in the distributed brain regions can provide information about the nature of diseases affecting the brain and the alterations of frequency specificity are associated with the pathological characteristics of mTLE.

## Introduction

Mesial temporal lobe epilepsy (mTLE) with hippocampal sclerosis (HS) is often accompanied by widespread alterations in structure and dysfunction over the entire brain [1]. With good spatial and temporal resolution, blood oxygenation level-dependent functional magnetic resonance imaging (BOLD-fMRI) is a promising approach for disentangling network structures and investigating the influences of network changes. Using BOLD-fMRI, the aberrant neuronal synchronization [2] and the impaired resting-state networks [3, 4] of mTLE have been detected. Furthermore, the different patterns of functional connectivity and hippocampal asymmetry between patients with left mTLE and those with right mTLE have been discussed in [5, 6].

The BOLD signal analysis technique provides an important means for the investigation of the pathological characteristics of mTLE. Previous studies have so far largely focused on characterizing the amplitude of the BOLD signals. Along this line, many analysis techniques have been explored, such as the two-dimensional temporal clustering analysis (2dTCA) [7], the regional homogeneity (ReHo) [8] and the functional connectivity based on linear correlation [9]. The disadvantage of these methods lies in that they do not work well in the cases where the amplitudes of the two oscillations are correlated but the underlying oscillations are neither phase coherent nor frequency consistent. Some studies suggested that the synchronization of oscillatory neuronal activity may play a key role in the integration of sensory signals during perceptual organization [10]. The frequency-specific correlated oscillations in the distributed brain regions may provide the indices of the network interactions that underlie cognitive processes [10]. Biswal et al. [9] suggested that the low frequency fluctuations of the BOLD signals in resting state reflect the spontaneous fluctuations in brain physiology and metabolism at baseline. The frequency content of the BOLD signals may contain useful information about the nature of diseases affecting the brain and the neurophysiological basis of mTLE. However, it remains unclear whether the different frequency specificity exists in the BOLD signals of patients with mTLE and healthy controls. Therefore, it is of great significance to investigate the frequency properties of BOLD signals in the setting of mTLE.

As regards the frequency analysis of BOLD signals, existing researches resort to dividing the whole bandwidth into the arbitrarily determined smaller frequency bins by using low-pass/band-pass filters and then estimating the Fourier power spectra or functional connectivity in each of the bins [11, 12]. Although these approaches have their own advantages in specific applications, their direct application to fMRI BOLD signal analysis might not be appropriate. For one hand, the traditional analysis techniques such as Fourier transform and Wavelet transform are only suitable for linear signal processing. However, fMRI BOLD signal is characterized by nonlinearity, non-stationarity and composition of signal components at multiple time scales [13]. On the other hand, the arbitrary division of the whole bandwidth cannot ensure that the intrinsic and biologically meaningful components rather than mathematically defined ones can be decomposed. Therefore, it is meaningful to explore the effective approach for the adaptive decomposition of the BOLD signals.

For the adaptive analysis of nonlinear and non-stationary fMRI signal, the well-known Hilbert-Huang transform (HHT) method [14] is a promising data-driven method. This method has been applied to clinical researches such as Parkinson’s disease and depression [15]. HHT adaptively decomposes a signal into a set of “intrinsic mode functions” (IMFs) using the empirical mode decomposition (EMD), and the instantaneous frequency of each IMF is derived from Hilbert transform. For the computation of the meaningful instantaneous frequency, this method requires that the signal itself is locally smooth and the derivative of the phase function exists. Unfortunately, the BOLD signal does not always conform to the hypothesis. In order to address this issue, the extreme-point symmetric mode decomposition (ESMD) method has been developed. Distinctively, this method utilizes the data-based direct interpolation (DI) approach to calculate the meaningful instantaneous frequency point by point in time [16].

In this paper, the ESMD is employed to investigate the frequency specificity of fMRI in mTLE. Different from the previous studies, our method automatically divides the whole frequency band into the adaptively determined sub-bands without any assumption of linearity or stationarity. Meanwhile, we compute the instantaneous frequency of each voxel using the DI approach for each subject, and then obtain its physiologically meaningful intrinsic frequency by the weighted sum of the instantaneous frequencies of all the IMFs where the square of instantaneous amplitude and the Euclidean norm of amplitude are used as the weight. Based on the assumption that the alterations of frequency specificity are associated with the pathological characteristics of mTLE, we use the intrinsic frequency value of each voxel as an important index of the BOLD signals and investigate the altered frequency specificity across different brain regions of the patients with mTLE by comparing them with healthy controls.

## Materials and Methods

### Subjects

Forty one right-handed patients were enrolled in this study. They were diagnosed with mTLE and unilateral hippocampal sclerosis (HS), including 17 patients with right mTLE (age: 22.2±9.6 years, durations: 6.3±5.8 years, 13 female) and 24 patients with left mTLE (age: 27.5±16.3 years, durations: 8.3±9.0 years, 13 female). All patients were screened using the strict inclusion/exclusion criteria: 1) Only the patients suffering from a complex partial seizure and who had one or more typical symptoms of mTLE-HS were included; 2) MRI was used to identify the epileptic foci on the conventional anatomical images whether the volume of unilateral hippocampus atrophy was significant or the unilateral hippocampal volume was less than the Chinese normal hippocampus volume. Increase in T2 fluid attenuated inverted recovery (FLAIR) signal in the hippocampus was used as the diagnostic criteria for HS; 3) At least two epileptiform discharges were detected predominantly (spikes, sharp waves, and/or spike-and-slow-wave complex) by scalp and sphenoidal EEGs on either left or right side of the hemisphere in the patients. The left-side or right-side seizure onset was further confirmed using the ictal video-EEG recordings. All the above evidences were employed for the identification of the side of mTLE.

Twenty four right-handed healthy volunteers were recruited as the controls (age: 31.5±12.9 years, 15 female). None of them had neurological or psychiatric disorders. Written informed consent was obtained from all subjects. The current research was approved by the ethics committee of Tongji Medical College, Huazhong University of Science and Technology, China.

### MRI Data Acquisition

MRI data were collected using a 3.0-Tesla MRI scanner (Siemens TRIO TIM, Erlangen, Germany), which was equipped with a 12-channel head coil at the Department of Radiology, Wuhan Union Hospital, China. Patients were examined during a seizure-free interval. The participants were instructed to rest with their eyes closed, keep their heads still, and not to fall asleep during the MR acquisition. A sagittal magnetization prepared rapid gradient echo three-dimensional (3D) T1-weighted sequence (T1 MPRAGE) was used to acquire the structural images with repetition time (TR) = 1900 ms, echo time (TE) = 2.26 ms, inversion time (TI) = 900 ms, Flip Angle (FA) = 9°, voxel size = 1×1×1 mm^3^, and 176 slices. A gradient echo-planar imaging (EPI) sequence was used for acquiring resting state functional images with TR = 2000 ms, TE = 30 ms, flip angle = 90°, matrix size: 64 × 64, voxel size = 3×3×3 mm^3^, slice thickness/gap = 3mm/1.2 mm, field of view = 200 × 200 mm^2^, and 32 slices. The scan for resting-state fMRI (RS-fMRI) lasted for 488 seconds for each subject, containing 240 brain volumes.

### Image Preprocessing

Resting-state fMRI data were preprocessed by using DPARSF [17] based on SPM8 (http://www.fil.ion.ucl.ac.uk/spm) and REST toolkits [18] (http://www.restfmri.net). The first 10 volumes of each participant were removed to eliminate the non-equilibrium effect of magnetization. The remaining 230 scans were slice-time corrected and then realigned to the first volume to correct head motion. Subjects with head motion greater than 2 mm or 2 degrees in any of the six parameters (*x*, *y*, *z*, pitch, roll, yaw) were excluded. All realigned images were spatially normalized to the Montreal Neurological Institute (MNI) template using the standard echo-planar imaging (EPI) template, thereby generating functional image series of 61×73×61 voxels (of size 3 mm×3mm×3mm). Then the data were smoothed with an isotropic 4 mm full width at half maximum (FWHM). Linear trend was regressed out from the time series of each voxel to remove the signal drifts arising from the scanner instability or other factors. A custom gray matter (GM) mask was created in SPM8 by thresholding the mean GM probability map from all individuals (threshold = 0.5). All subsequent analyses were restricted to the custom grey matter mask. This procedure was implemented to exclude the cofounding effect from the cerebrospinal fluid (CSF) and white matter (WM) signals. No temporal filtering was implemented during the preprocessing to ensure that the signal analysis could be performed within full frequency realm (0–0.25 Hz).

### Extreme-point Symmetric Mode Decomposition

In the ESMD method, the signal will be firstly decomposed into a series of IMFs together with an optimal adaptive global mean (AGM) curve. Then, the DI approach is applied to each IMF to yield the instantaneous amplitudes and frequencies together with a time-varying energy [16]. For the original BOLD signal *Y*, it can be composed using the ESMD method as:

$$Y=\sum_{j=1}^{M}{\text{IMF}}_{j}+R$$ (Eq 1)

where *M* is the number of IMFs, *R* is the final residue which has been optimized to be an optimal AGM curve with a certain number of extreme points in the process of decomposition.

Considering that the original BOLD signal *Y* and the residue signal *R* are discrete time signal after sampling, we denote the two signals by *Y =* {*y*_*i*_*|*1≤*i*≤*N*} and *R =* {*r*_*i*_*|*1≤*i*≤*N*}, where *N* is the number of time points. For the two signals, the corresponding variances will be given by:

$$\sigma_{0}^{2}=\frac{1}{N}\sum_{i=1}^{N}{\left(y_{i}-\bar{Y}\right)}^{2}$$ (Eq 2)

$$\text{with}\;\bar{Y}=\frac{1}{N}\sum_{i=1}^{N}y_{i}$$ (Eq 3)

$$\sigma^{2}=\frac{1}{N}\sum_{i=1}^{N}{\left(y_{i}-r_{i}\right)}^{2}$$ (Eq 4)

Inspired by the reference [16], the specific decomposition of the BOLD signals using the ESMD method is described as follows:

Step 1: Find the locations of all the extrema of *Y*, connect all the adjacent extreme points with line segments, mark their midpoints, and add the left and right boundary midpoints;

Step 2: Construct *p* interpolation curves with all these midpoints *L* (*L =* {*L*_*i*_*|*1≤*i*≤*p*}) and calculate their mean value as:

$$L^{*}=\frac{\sum_{i=1}^{p}L_{i}}{p}$$ (Eq 5)

Step 3: Repeat the above two steps on *Y*-L* until the absolute value of L* is less than a permitted error *ε* (*ε* = 0.001σ_0_) or the sifting times attains a preset maximum value *K*. Then get the first mode IMF_1_.

Step 4: Repeat Steps 1–3 on the residual *Y-*IMF_1_ and obtain all other IMFs until the number of extreme points in the last residual *R* is less than the predefined constant *S*;

Step 5: Change the maximum number *K* within a finite integer interval from *K*_min_ *to K*_max_ and repeat the above four steps. Then calculate the ratio ν of variance (ν = σ/σ_0_) for different *K*;

Step 6: Find the optimal sifting times *K*_0_ corresponding to the minimum value of ν in the range from *K*_min_ to *K*_max_. Then use this *K*_0_ to repeat the process from Step 1 to Step4 and output all the IMFs as well as the last residue *R*, which is actually the optimal AGM curve.

### Frequency Specificity Analysis

Based on the obtained IMFs of the original BOLD signals, the meaningful instantaneous frequency at each time point can be yielded by the direct interpolation of the ESMD. The detailed implementation of the direct interpolation involves the following three steps [16]:

Step 1: Extract the extrema of each IMF. Calculate the time interval between each pair of adjacent maxima as the local period. Obtain the local period for the adjacent minima in the same way.

Step 2: Obtain the local frequency for the midpoint of the adjacent maxima by the reciprocal of the local period. Implement the similar procedure for the midpoint of the adjacent minima. Add the left and right boundary points using the linear interpolation method.

Step 3: Implement cubic spline interpolation with all the discrete points and get the instantaneous frequency *f*(*i*) at time point *i*. Please note that the instantaneous frequency is defined as max{0, *f*(*i*)} to ensure that *f*(*i*) is meaningful.

Inspired by the work of Xie et al. [19], the mean frequency *Freq*(*j*) for *IMF*_*j*_ is computed as:

$$Freq(j)=\frac{\sum_{i=1}^{N}f_{j}(i)a_{j}^{2}(i)}{\sum_{i=1}^{N}a_{j}^{2}(i)}$$ (Eq 6)

where *f*_*j*_(*i*) is the instantaneous frequency of *IMF*_*j*_ at the time point *i*, *a*_*j*_(*i*) is the instantaneous amplitude of *IMF*_*j*_ at the time point *i*. The square of instantaneous amplitude is used as weight as suggested in [19]. Here, *Freq*(*j*) reflects the mean oscillation frequency of the IMF, and thus it is a proper measure of the mean frequency of the signal in the narrow frequency band.

By the weighted sum of the mean frequency of all IMFs, the intrinsic frequency *Freq* of the original BOLD signals is calculated as [19]:

$$Freq=\frac{\sum_{j=1}^{M}\left\|a_{j}\right\|Freq(j)}{\sum_{j=1}^{M}\left\|a_{j}\right\|}$$ (Eq 7)

where ‖*a*_*j*_‖ denotes the Euclidean norm of amplitude *a*_*j*_. The intrinsic frequency *Freq* can be in analogy to the eigenfrequency of objects in the field of physics in the unit of Hertz.

We use the ESMD approach to analyze the fMRI resting-state data of three groups including 65 subjects. The intrinsic frequency *Freq* for every voxel in the brain of the individual subject is calculated. These values across subjects of each group are averaged to reveal the whole frequency distribution for BOLD oscillations of all subjects. Group-averaged statistical histograms of frequency distributions are used to indicate the spatial distribution features in the whole brain. The statistical histograms related to frequency distributions of bilateral hippocampus across the patients with mTLE and the healthy controls are obtained to reveal the hemispheric asymmetry of the human hippocampus. To observe which brain regions have the significantly higher *Freq* value than the global mean in each group, we perform the one-sample *t*-test to generate the *T* maps for both patient and healthy control groups. The voxel-wise two sample *t-*test has also been performed to compare the *Freq* results between patients and controls using the statistical analysis tool in REST software based on SPM8. For the one-sample *t*-test, a single voxel threshold is set at *p* < 0.05 and a minimum cluster size of 2160 mm^3^ is used to correct for multiple comparisons using false discovery rate (FDR) correction (*Q*-value <0.01). For the two-sample *t*-test, a single voxel threshold is set at *p* < 0.05 and a minimum cluster size of 2295 mm^3^ is used to correct for multiple comparison using AlphaSim. We attempt to find the differences of *Freq* distributions between patients with mTLE and healthy controls. These differences will provide a justification and indication for epilepsy diagnosis and pathological study.

## Results

**Fig 1:**
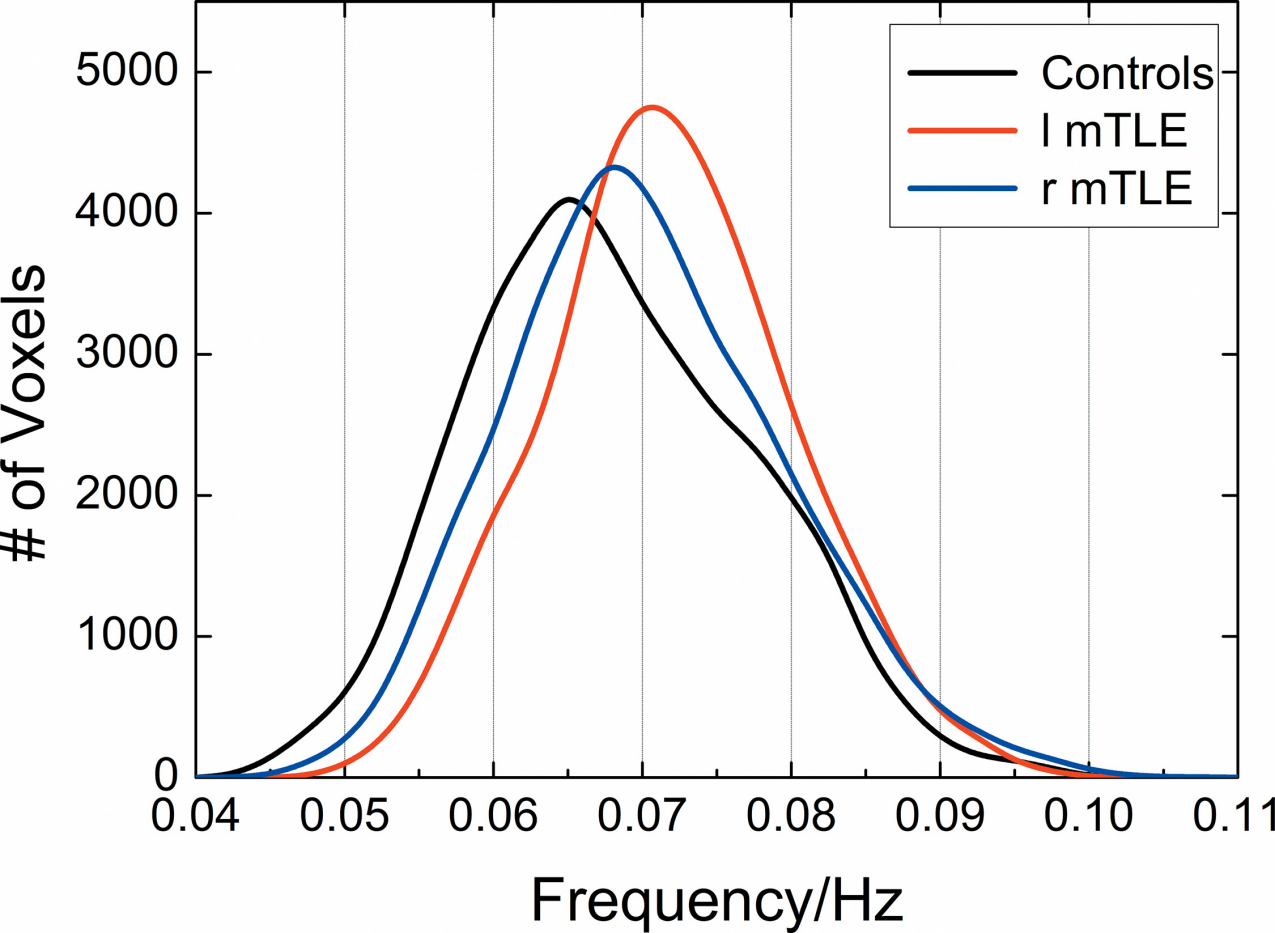
Histograms of the whole-brain *Freq* distribution across patients with mTLE and healthy controls. The histograms are measured only for gray matter voxels in the entire brain and averaged across all the subjects in each group. Height of the histograms represents the number of voxels whose *Freq* is equal to the frequency on the horizontal axis. The healthy controls, patients with left mTLE and those with right mTLE are color-coded by black, red and blue, respectively.

The statistical histograms for *Freq* distributions of the whole-brain voxels across patients with mTLE and healthy controls are shown in **Fig 1**. The frequencies of all the voxels in the brain range from 0.04 to 0.11Hz with a skewed normal distribution. For healthy controls, patients with right mTLE and those with left mTLE, the peaks appear at 0.065Hz, 0.068Hz and 0.07Hz, respectively. Most voxels are distributed around the peak frequency. Although there are a lot of overlap in *Freq* distributions between patients with mTLE and healthy controls, it is obvious that the peak of the curve for the patients moves towards the higher frequency than that for the healthy controls. Meanwhile, the patients with left mTLE have higher peak frequency than those with right mTLE. The mean and standard deviation of *Freq* across all subjects are 0.0679±0.0095 (controls), 0.0718±0.0082 (left mTLE), and 0.0702±0.0093 (right mTLE). In addition, a nonparametric test among the three groups was performed by using SPSS (http://www-01.ibm.com/software/cn/analytics/spss/). The results of Moses Extreme Reactions test demonstrate that the differences in frequency distributions between patients with mTLE and controls are statistically significant (*p* < 0.01). These frequency properties make it possible for us to establish a link between epilepsy pathology and the intrinsic frequency *Freq* of patients with mTLE.

**Fig 2:**
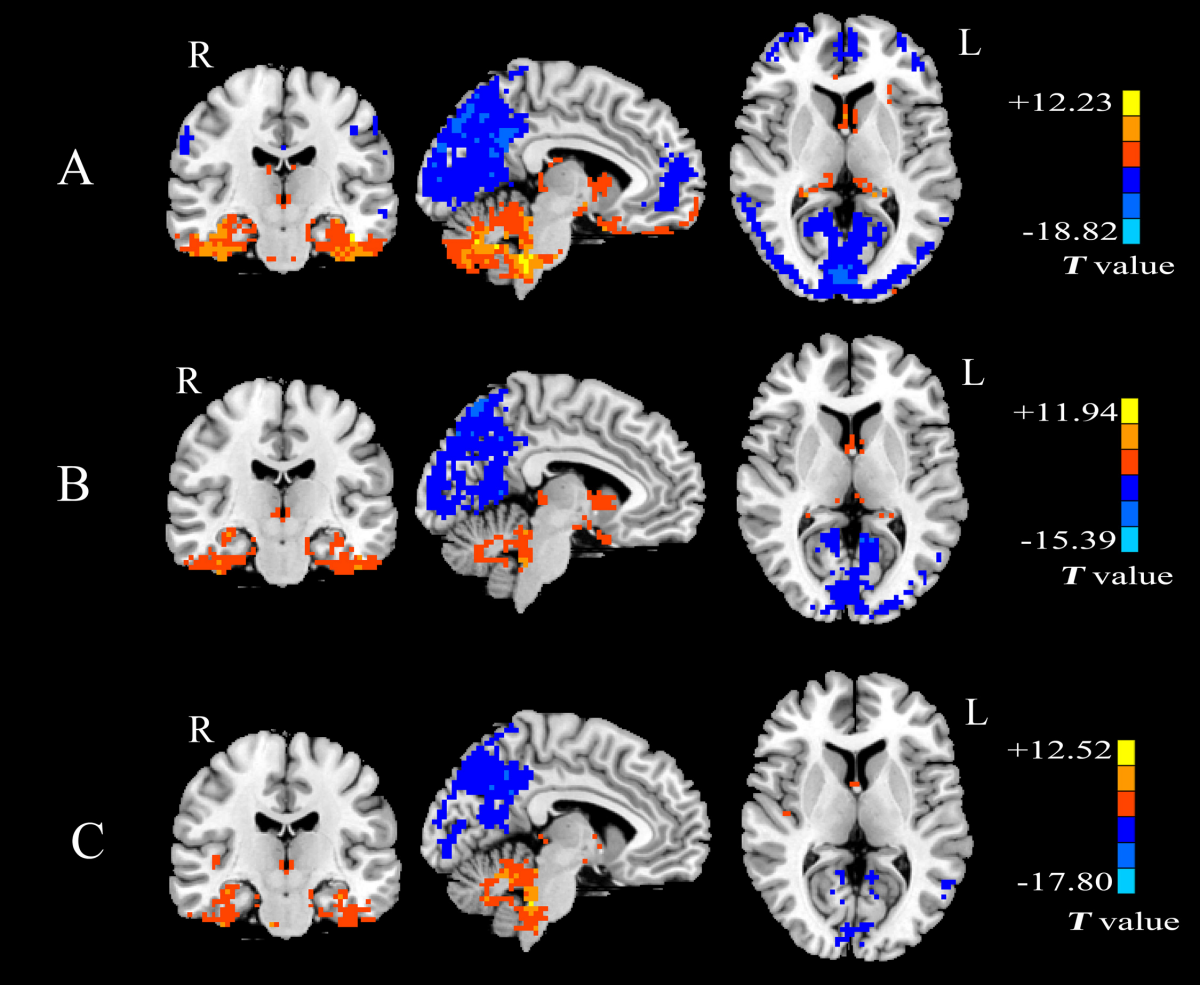
Results of the one sample *t*-test for *Freq* map across the three groups. (A) healthy controls. (B) patients with left mTLE. (C) patients with right mTLE. One sample *t*-test, *p* < 0.05, voxels > 80, with FDR correction (*Q* value < 0.01). Color scale indicates the high *Freq* value.

**Fig 2**shows the results for the one-sample *t*-test. The brain regions with higher and lower *Freq* than the global mean value can be easily observed from **Fig 2**for both healthy controls and patients with mTLE. In the healthy control group, a significantly lower *Freq* is found mainly in the default mode network (DMN) (including precuneus, bilateral inferior parietal lobule, posterior cingulate cortex (PCC), medial prefrontal cortex (MPFC)), frontal lobe and bilateral calcarine gyrus. Whereas, a significantly higher *Freq* is found mainly in the bilateral parahippocampa gyrus, superior temporal gyrus, inferior temporal gyrus, insula and the cerebellar region as shown in **Fig 2A**. In the patient group, the lower *Freq* is observed mainly in precuneus, PCC and bilateral calcarine gyrus while the higher *Freq* is concentrated in the inferior temporal gyrus and the cerebellum as shown in **Fig 2B and 2C**, respectively. However, compared with the healthy controls, the patients show a decrease in the size of above-mentioned regions. The most prominent difference is that no significantly lower *Freq* has been observed in the MPFC for the patient group, which indicates that the *Freq* value of MPFC is equal to the global mean value.

**Fig 3:**
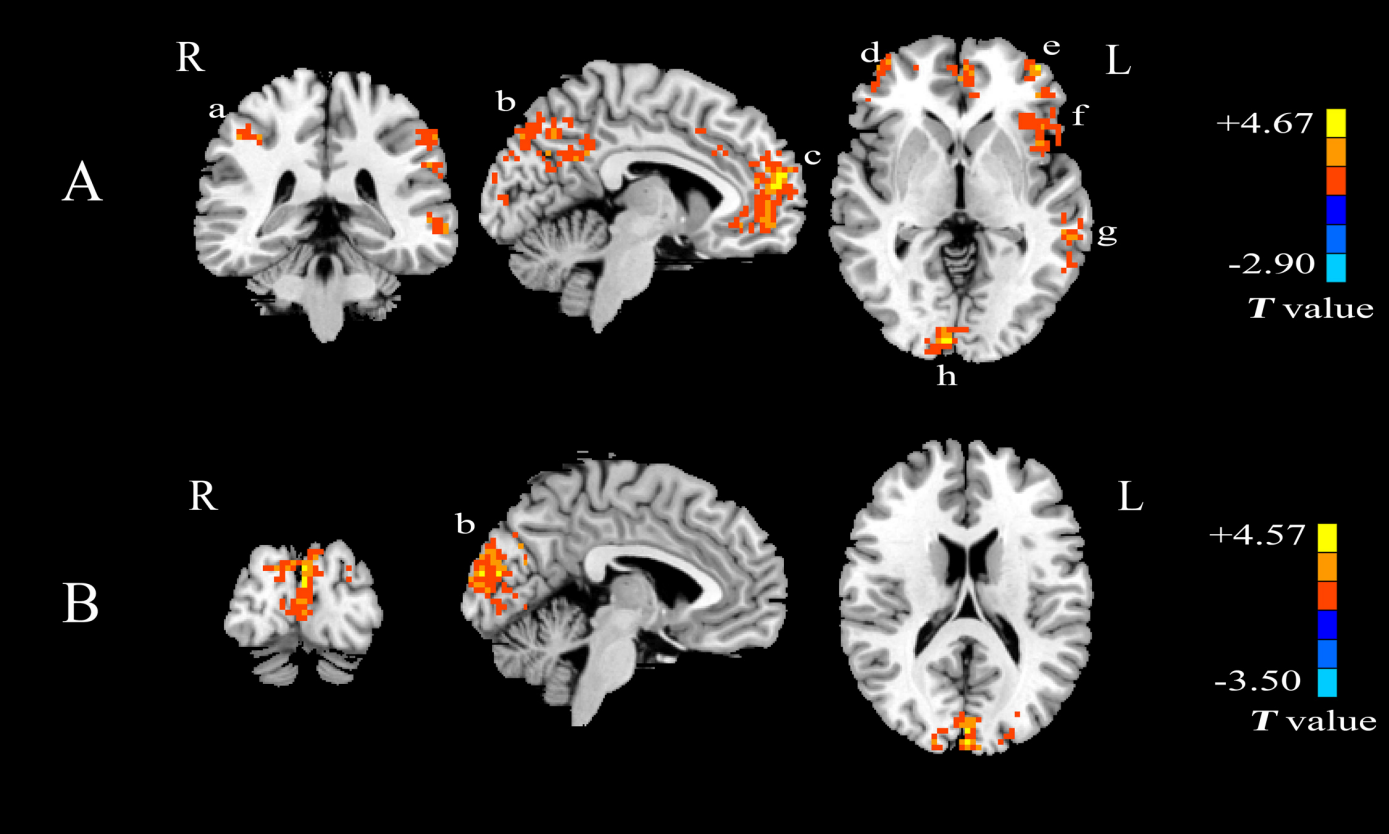
Statistical *T*-map showing the differences between patients with mTLE and healthy control. (A) Comparison of *Freq* between patients with left mTLE and controls; (B) Comparison of *Freq* between patients with right mTLE and controls. Two sample *t*-test, *p* < 0.05, voxel > 85, with AlphaSim correction. Warm colors indicate that the *Freq* of patients with mTLE is higher than that of healthy controls whereas cool colors have the opposite meanings. The areas are labeled: a. bilateral inferior parietal lobule(IPL), b. posterior cingulate cortex (PCC)/ precuneus, c. medial prefrontal cortex (MPFC), d. right middle frontal gyrus (MFG), e. left middle frontal gyrus(MFG), f. insula, g. middle temporal gyrus (MTG), h. calcarine gyrus.

**Fig 4:**
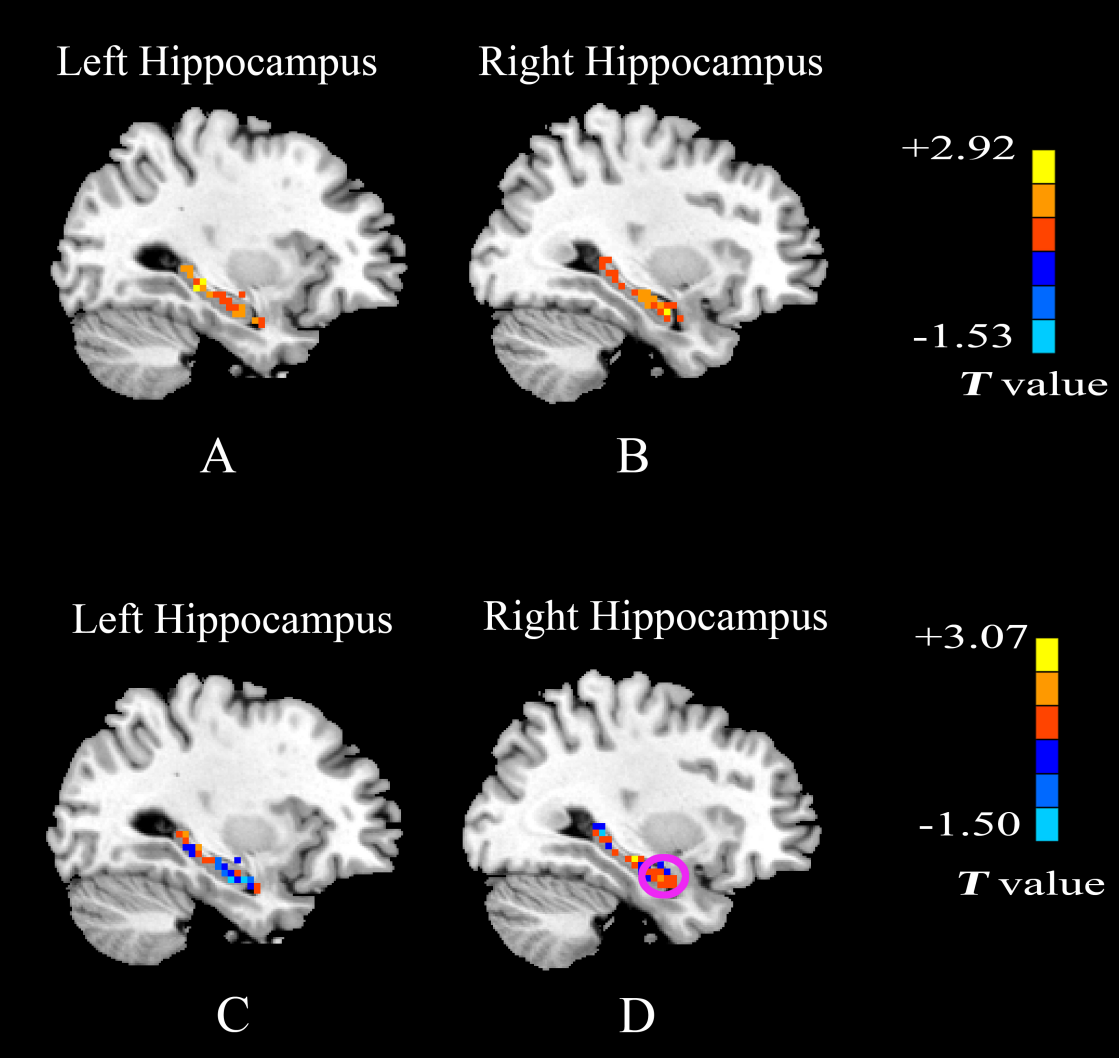
Statistical *T*-map showing the differences in the bilateral hippocampus between patients with mTLE and healthy controls. Results of the two sample *t*-test without correction. (A) and (B) Comparison of *Freq* in the bilateral hippocampus between patients with left mTLE and controls; (C) and (D) Comparison of *Freq* in the bilateral hippocampus between patients with right mTLE and controls. The meanings of colors are the same to those in Fig 3.

The two-sample *t*-test has also been performed to examine the differences between patients with mTLE and healthy controls. **Figs 3 and 4**show the comparisons of *Freq* within the whole brain and *Freq* in the bilateral hippocampus between patients with mTLE and controls, respectively. **Table 1**lists the regions showing significantly higher *Freq* for patients with mTLE. It can be seen from **Fig 3**that compared with the controls, the group with left mTLE shows a higher *Freq* in the precuneus, PCC, MPFC, bilateral inferior parietal lobule (IPL), middle frontal gyrus (MFG), insula, middle temporal gyrus (MTG), calcarine gyrus and the cerebellar region **(Fig 3A)**. No significantly lower *Freq* has been observed. In comparison to healthy controls, the group with right mTLE shows a higher *Freq* in the precuneus, occipital lobe and no significantly lower *Freq* has been found **(Fig 3B)**. The differences between patients with mTLE and healthy controls within the bilateral hippocampus are shown in **Fig 4**(no correction). It is noticed that patients with left mTLE show higher *Freq* in the bilateral hippocampus **(Fig 4A and 4B)** while patients with right mTLE only show higher *Freq* in the affected right hippocampus **(Fig 4C and 4D)**, especially in the anterior section (marked with a circle).

**Table 1:** Regions Showing Significantly Higher *Freq* in Patients with mTLE.

Brain regions	H	Epileptogenic zone laterality	Peak MNI coordinate			Cluster Voxels	Peak *t* value
			X	Y	Z		
Calcarine gyrus, precuneus,PCC	R/L	Left	12	-96	-3	700	4.32
Middle temporal gyrus	L	Left	-63	-12	-9	242	3.80
Inferior frontal gyrus(L)^a^, insula	R/L	Left	-48	42	6	1073	4.66
Middle frontal gyrus	R	Left	42	51	15	169	4.67
Inferior parietal lobule	R/L	Left	39	-66	42	209	3.74
Mesial prefrontal cortex	R	Left	3	57	9	70	4.04
Mesial prefrontal cortex	L	Left	-3	57	9	78	4.26
Precuneus	R/L	Right	0	-90	18	343	4.47

Abbreviations: mTLE, mesial temporal lobe epilepsy; H, hemisphere; PCC, posterior cingulate cortex; R, right; L, left. Two-sample *t*-test, *p* < 0.05, voxel > 85, using AlphaSim correction.

^a^ represents that superior frontal gyrus and middle frontal gyrus are also with significant higher *Freq* in addition to inferior frontal gyrus.

To determine the impact of mTLE seizures on hippocampal frequency specificity, the mean *Freq* values and standard deviation of the bilateral hippocampus for healthy controls and patients with mTLE are calculated and shown in **Table 2.** On the whole, the patients show higher mean *Freq* than the controls in the bilateral hippocampus. In the healthy controls, the mean *Freq* of left hippocampus is higher than that of right hippocampus. In the patients with mTLE, a higher mean *Freq* occurs in the affected ipsilateral hippocampus than in the unaffected contralateral hippocampus. The results of significance test (Manne-Whitney test) between healthy controls and patients with mTLE within the bilateral hippocampus are shown in Table 2. The differences between the following four pairs of groups are statistically significant (corrected *p* < 0.001), healthy controls vs patients with left mTLE in the bilateral hippocampus, healthy controls vs patients with right mTLE in the right hippocampus, patients with left mTLE vs patients with right mTLE in the left hippocampus. No statistical differences were observed in the other two groups, healthy controls vs patients with right mTLE in the left hippocampus, and patients with left mTLE vs patients with right mTLE in the right hippocampus.

**Table 2:** Mean *Freq* Values and Standard Deviation of the Bilateral Hippocampus and the Results of Significance Test for Healthy Controls and Patients with mTLE.

ROI	Controls Mean (SD)	l mTLE Mean (SD)	r mTLE Mean (SD)	Control–l mTLE Sig.	Control–r mTLE Sig.	l mTLE–r mTLE Sig.
Left Hippocampus	0.0803 (0.0039)	0.0823 (0.0037)	0.0802 (0.0041)	*p* < 0.001^a^	NS^b^	*p* < 0.001^a^
Right Hippocampus	0.0793 (0.0034)	0.0820 (0.0036)	0.0816 (0.0039)	*p* < 0.001^a^	*p* < 0.001^a^	NS^b^

Abbreviations: SD, Standard Deviation; mTLE, mesial temporal lobe epilepsy; Sig, Statistical significance

^a^ represents that Manne-Whitney test is performed, *p* < 0.001.

^b^ represents that there are no significant differences between the two groups.

**Fig 5:**
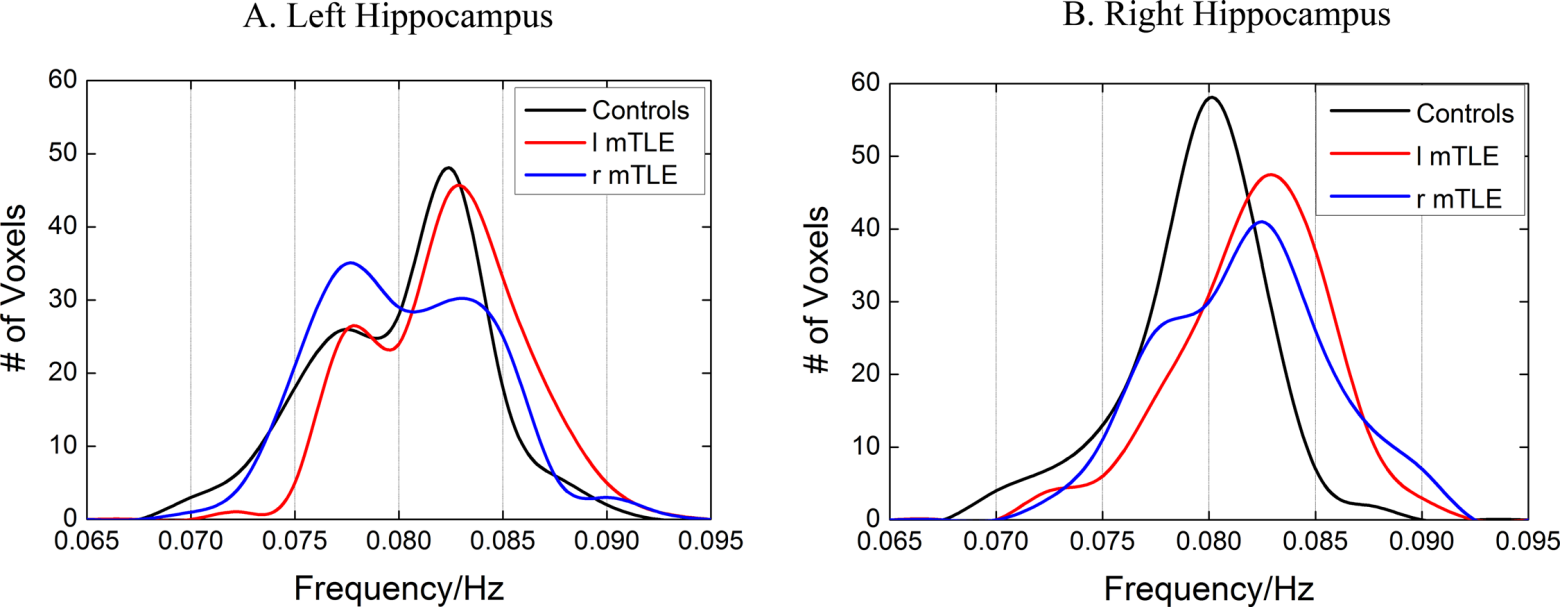
Histograms of the *Freq* distribution of the unilateral hippocampus across patients with mTLE and healthy controls. The healthy controls, patients with left mTLE and those with right mTLE are color-coded by black, red and blue, respectively. (A) left hippocampus. (B) right hippocampus. The histograms are measured only for gray matter voxels and averaged across subjects in each group. Height of the histograms represents the number of voxels whose *Freq* equals the frequency on the horizontal axis.

The statistical histograms for *Freq* distributions of the bilateral hippocampus across patients with mTLE and healthy controls are shown in **Fig 5.** In comparison to the frequency scope of the whole-brain voxels, the *Freq* of the voxels in the bilateral hippocampus involves a narrower frequency range and is dominated by higher-frequency BOLD oscillations (>0.065Hz). It is remarkable that the distribution curve of the left hippocampus in three groups shows two peaks (**Fig 5A**) while that of the right hippocampus only has a single peak (**Fig 5B**). These findings may provide new evidence for the asymmetry of anatomical and functional correlates of human bilateral hippocampus. It is noticed that the peak of the curve for patients with left mTLE moves towards a higher frequency in the bilateral hippocampus while such a phenomenon only occurs in the affected ipsilateral hippocampus (right) for patients with right mTLE.

## Discussion

In the present fMRI study, a data-driven approach ESMD has been employed to measure the intrinsic frequency of the BOLD-fMRI signals and demonstrate the alterations of frequency specificity in the patients with mTLE. Here, these findings will be discussed in more details.

The spatial frequency distributions for the three groups are skewed normal distributions, which are approximately symmetrical at the mean of the data as shown in Fig 1. However, the distribution pattern for the patients with mTLE significantly differs from that for the healthy controls. The peak of the curve for the patients moves towards the higher frequency than that for the controls. In addition, the patients show higher mean *Freq* than the controls. The spread of the distribution for the patients with mTLE is narrower than that for the controls because the standard deviation of *Freq* for the patients is smaller than that for the controls. On the whole, the patients with left mTLE show more obvious alterations in the peak of the curve and mean *Freq* than those with right mTLE. The shift of distributions and higher mean *Freq* for patients with mTLE can be attributed to the appearance of higher *Freq* in some brain regions which may be related to the structural and functional abnormalities of these patients. Furthermore, we hypothesized that the higher the *Freq*, the more severe the clinical symptoms. Hippocampal sclerosis is the most common clinical symptom in mTLE. This symptom is associated with the asymmetrical extra-hippocampal gray matter loss which can encompass the ipsilateral and contralateral hemisphere, particularly the contralateral hippocampus, more pronounced in patients with left mTLE [20]. This result is consistent with the finding of the more obvious alteration in patients with left mTLE in the present research. Moreover, it is likely that the interictal epileptic activity, just like a goal-directed task, may induce the BOLD deviations, which can enhance the value of *Freq* and lead to the narrower spread of the distribution for patients. This assumption can be supported by some previous findings [21, 22] that compared with the resting-state, BOLD trajectories become more constrained and the power distribution across frequencies becomes narrower during task-state.

The one sample *t*-test **(Fig 2A, top row)** shows that in the healthy control group, DMN, frontal lobe and bilateral calcarine gyrus are the main areas which show significantly lower *Freq* while the significantly higher *Freq* has been found mainly in the bilateral parahippocampa gyrus, superior temporal gyrus, inferior temporal gyrus and insula. The test results agree with the findings from Baria et al. [11] to some extent. In their work, it was reported that the lowest-frequency band was localized mainly in prefrontal and occipital cortex. The higher-frequency bands were located more within cingulate, insula, temporal cortex, and subcortical structures. Indeed, Baria’s findings corroborate the frequency analysis results in our study.

The results of the one sample *t*-test **(Fig 2B and 2C)** show that the reduced area in DMN and absence of MPFC are the main characteristics in patients with mTLE. Moreover, group comparisons with the two sample *t*-test confirm that the higher *Freq* appears in the DMN of patients with left mTLE and in precuneus of patients with right mTLE (**Fig 3**and **Table 1**). Such findings can be explained further. It has been verified that the DMN, as one of the most important resting-state networks, assists memory consolidation, task engagement and response preparation [23, 24]. Increasing evidences from behavioral and neuroimaging studies suggest that mesial temporal lobe epilepsy is possibly associated with the function of DMN [25] and the aberrant DMN regions are commonly considered to be involved in the mesial temporal epileptic network [26–29]. Meanwhile, by applying graph-theoretic concepts to Granger causality analysis, it has been proven that MPFC is the second key hub of the DMN driven by the other nodes and the precuneus cortex is the primary target of causal influences [30]. Based on these studies, we speculate that the higher *Freq* in the DMN of patients with mTLE may reflect the functional impairment within the DMN arising from the focused lesion of the seizure.

The results of the two sample *t*-test (**Fig 3**and **Table 1**) also show a higher *Freq* in the hippocampus, insula and middle temporal gyrus in the group with mTLE than in the healthy control group. We infer that the hippocampus is an epileptogenic zone in the mTLE and the insular cortex plays an essential role in seizure propagation in patients with mTLE. The neuroimaging and positron emission tomography (PET) studies [31], [32] support such an inference. Meanwhile, mesial temporal lobe is known to be highly involved in episodic memory [33] and the disruption [34] of neural circuitry in mTLE. Furthermore, it is found that the cerebellum posterior lobe shows a higher *Freq* in the group with left mTLE than in the healthy control group. Because one of the main functions of cerebellum is motor coordination [2], it is likely that higher *Freq* could be a sign of decreased motor coordination.

For the hemispheric asymmetry of the human hippocampus, several advancements have been achieved in the study of hippocampal structural and functional asymmetries [35] while the molecular basis of hippocampal asymmetry is still on the way. Woolard et al. [36] found that the degree of anterior hippocampal volume asymmetry predicted the performance about the measure of basic cognitive abilities. In our study, the following findings provide evidence for the regional specificity and functional implications of the well-known hemispheric asymmetry of hippocampus. **Fig 4**and **Fig 5**demonstrate the difference of the frequency distribution between the left hippocampus and the right hippocampus. The left hippocampus shows two peaks while the right hippocampus shows only a single peak. In the healthy controls, the mean *Freq* of the left hippocampus is higher than that of the right hippocampus. The *Freq* distribution curve in the bilateral hippocampus of patients with left mTLE moves towards the higher frequency than that of healthy controls, while this phenomenon only appears in the right hippocampus of patients with right mTLE. In comparison to the healthy controls, the patients with left mTLE show the obvious alteration in the bilateral hippocampus, while the patients with right mTLE only shows the difference in the affected right hippocampus (**Table 2**). It is interesting that our observation is similar to the result of several neuroimaging investigations. Vannest et al. [37] reported that patients with right hemisphere epilepsy showed an insignificant increase in the degree of left lateralization. In contrast, the patients with left hemispheric epilepsy significantly differed from the controls and patients with right hemispheric epilepsy in that the former showed right-lateralized activation. During the encoding of verbal stimuli task, Wagner et al. [38] found that hippocampal activation patterns in patients with left TLE were more right-lateralized than those with right TLE. Similarly, functional connectivity resulted in more ipsilateral damage to the seizure focus in patients with mTLE, and the left hippocampal sclerosis caused more reduction of functional connectivity than the right one. All these evidences emphasize a distinct role of the left and right hippocampus in the brain organization and suggest that different approaches have to be used to deal with patients with left and right mTLE.

In summary, the frequency-specific distributions of resting state fMRI data obtained by the ESMD method indicate that the alterations of frequency specificity are associated with the pathological characteristics of mTLE. These findings provide the additional evidence to support epileptogenic network theories [2]. Based on the *Freq* values measured in this study, we propose that the DMN, middle frontal gyrus, insula, middle temporal gyrus, calcarine gyrus and hippocampus with the abnormally higher *Freq* comprise a network, which might be involved in the generation and propagation of epileptiform activity. The abnormally higher *Freq* in such regions as specifically DMN, the visual cortex (calcarine gyrus) and cerebellum might suggest a widespread functional impairment through a remote effect of seizure lesion within the mesial temporal epileptic network. The distinct frequency-specific characteristics in the left hippocampus and the right hippocampus may reflect the hippocampal structural and functional asymmetries. The distinct frequency patterns of the left mTLE and the right mTLE indicate that there are different pathological mechanisms underlying these two types of epilepsies.

## Conclusions

In this paper, we have proposed an effective ESMD based frequency specificity analysis method for the BOLD signals of mTLE. With this method, the fMRI signals are decomposed into several different frequency components. Based on these components, the intrinsic frequency *Freq* for each voxel of the human brain is calculated and then used as an indicator of the BOLD signals. By means of *Freq*, the one-sample and two-sample *t*-tests have been performed to investigate the altered frequency specificity across different brain regions of the patients with mTLE. Our results show that the areas with the consistently higher *Freq* may be ones which are responsible for seizure genesis and propagation or may correspond to wide functional impairments found in patients with mTLE. Besides, the hippocampal structural and functional asymmetries are also proved by the distribution curve of *Freq*. The preliminary results indeed indicate that the frequency specificity is associated with the pathological characteristics of mTLE although the fundamental mechanism between them remains subject of debate.
